# Items

**Published:** 1882-02

**Authors:** 


### Items.

---

Medical Examinations for the Army.—From the Annual report of Surgeon-General Barnes, the following extract is made :

The following is a recapitulation of the work thus far performed by the Army Medical Examining Board:

Number of Assistant Surgeons examined for promotion........................................44
Number of Candidates for appointment in the Medical Corps invited to appear for examination........................................221
Number of Candidates found qualified........29
" " " rejected...........47
" " " who withdrew after partial examination.......................107
Total number examined.......183
Number of Candidates who failed to appear for examination...........................17
Number of Candidates who declined to appear for examination.......................20
Number of Candidates remaining to be exined.................................1
Total number invited but not examined.......38

---

From the third Annual report of the Board of Health, of the City of Atlanta, for the year 1881, we learn: That the total number of deaths in this city for the year was 962—white, 423 ; colored, 539—against 679, for 1880.

Annual mortality per thousand 24.66. White, 19.22. Colored 31.70.

Consumption caused 104 deaths—white, 51; colored, 53. Pneumonia 67—white, 18; colored, 49. Other acute lung diseases 20. White and colored, each 10. Diarrhœal diseases 205—white, 92; colored, 113. Typhoid and "typho-malarial" fevers, 68—white, 29; colored, 39. Malarial fevers, 8—white, 5; colored, 3. Scarlet fever, 7—white, 6; colored, 1. Diphtheria, 3—white, 2; colored, 1. Rheumatism, 3, all colored. Thirty-seven deaths due to accident, 13 white, and 24 colored; and 2 suicides, both white, are not included. The removal of night-soil by wheel carriage works satisfactorily. Not a cess-pool or privy-vault exists in the city. The sewers are inadequate and some are poorly constructed and of improper material. The water supply is insufficient—though this will be remedied by spring. General vaccination, recently undertaken, is progressing well.

---

The Board of Pharmaceutic Examiners of Georgia met in this city December 13, 1881. One hundred and ten licenses were granted to applicants under the new law. Of this number one hundred and four were issued to persons holding diplomas, or who were otherwise exempted from personal examination under section 5 of the act.

Sixteen persons applied for personal examination and license—six passed and were granted licenses. Ten failed to attain the accepted standard and were refused licenses. The Board adopted the opinion of the Attorney General to the effect that delinquents should be prosecuted by the solicitors general of the State upon information furnished by the Board. The Board will hold its next meeting in Macon, for the examination of applicants and diplomas, on the third day of next April.

---

What a Gunshot Wound did for Science Half a Century Ago.—This is the title of a paper in the *College and Clinical Record*, which embraces a letter from Robley Dunglison, M.D., bearing date of Feb. 5th, 1833. It was written from the University of Virginia. It was a private communication to Dr. Isaac Hays, of Philadelphia, and afterwards published, in 1835, in the writer's work on hygiene. This gave an account of the wounding, and subsequent experiments upon, Alexis San Martin, of Canada, under charge of Dr. Beaumont. The man was wounded, we believe, in 1825, or earlier. He died last year. The profession is familiar with the services rendered science by his accident.

---

THE initial number of the *Independent Practitioner*, under its new management, has reached us. It is now edited by Basil M. Wilkerson, D.D.S., M.D., George H. Rohe, M.D., and George W. Fields, D.D.S. We extend most cordial greeting to our friend, Dr. Rohe, upon his entrance into this new field of activity and usefulness, and congratulate the *Practitioner* upon his acquisition. The *Practitioner* is determined, it says, to secure the recognition of dentistry as a medical specialty, if its earnest and honest efforts in this cause prevail. It is animated, so it would seem, by the same sentiment which was sustained at the late meeting of the International Medical Congress in London.

---

THE New York letter of the *Chicago Medical Examiner* says the number of students in the colleges of that city is somewhat smaller than the year before. Bellevue has regained to some extent the loss of size of class it experienced by attempting the three-year's course. It is a source of regret that the class diminished, in the first place; in the second, that the college so quickly gave heed to the whispers of expediency; and in the third place, that the profession gave the whispers (by withholding patronage) so readily. But the profession means well; and a little persistence on the part of Bellevue would have demonstrated it.

---

SAVANNAH has formed a "Citizens Sanitary Association." Every member, upon payment of regular dues, is entitled to report of the sanitary engineer, employed by the organization, as to the condition of premises, and plans for improvement if necessary; supervision of alterations in dwelling; annual inspection; occasional supplementary inspection and reports of officers. If properly managed—and its organization promises that it will be—it may become very popular, and it can undoubtedly accomplish much good in promoting public sanitation.

---

Ergot.—Dr. Ingals, president of the Chicago Medical Society, in an article (*Med. Jour. and Examiner*), on "The Management of Parturition," says: "Ergot seems to be a hæmostatic, but I have not much confidence in its power to excite uterine contractions, and I think it is best that we should place little reliance on it for the purpose." Now, this may seem somewhat positive, but we believe that before many years both the physiological and therapeutical action of ergot (as now recognized), is destined to undergo an overhauling.

---

Medical Knowledge.—In the address at the dedication of the new hall of the Boston Medical Library Association, the president, Oliver Wendell Holmes, said: "Our American atmosphere is vocal with the flippant loquacity of half knowledge. We must accept whatever good can be got out of it, and keep it under as we do sorrel and mullein and witchgrass, by enriching the soil, and sowing good seed in plenty; by good teaching and good books, rather than by wasting our time in talking against it. Half knowledge dreads nothing but whole knowledge."

---

A Learned Medical Body recently delivered itself in this wise. *Resolved*, That the Eclectic Medical Association of Connecticut hereby declares its convictions against the practice of vaccination, and all legislation making it compulsory; and asks the legislatures of the several States to pass laws prohibiting sanitary boards, school boards, and other local authorities from making and enforcing regulations for that purpose!

This is progress; anti-mineral progress. Let it proceed.

THE *Louisville Med. Herald* says: "The profession of Louisville has been excited of late over the prospective new summer school, and we went so far as to copy for our readers the statements of the daily press about the material out of which the faculty of the new candidate was to be made up. After a month's delay and a great deal of inquiry, we have learned that the sources of our information were not reliable, and that the profession here is not pregnant with anything like a new college."

---

THE ATLANTA MEDICAL SOCIETY was organized January 9th. Dr. W. S. Armstrong was elected President, and Dr. W. D. Bizzell Secretary. The objects of the organization are the promotion of professional interests; the upholding of medical ethics; the advancement of medical science; the cultivation of professional acquaintanceship and mutual improvement. This new society gives promise of much usefulness, and we confidently expect to see it accomplish great things in this inviting but unoccupied field.

---

THE *New England Medical Monthly* enjoys the distinction of being the handsomest medical periodical published in America—and, so far as we know, in the world.

It is always greeted, upon our table, with sincere interest, but it should not persist in confounding—orthographically, at least—this enterprising city and growing metropolis with a certain celebrated and swift-footed huntress of mythology.

---

ANOTHER MEDICAL COLLEGE.—This time it struck Baltimore. Prof. Harvey L. Byrd, formerly of Savannah, is President and Professor of Obstetrics and Diseases of Women and Children. Dr. Byrd, years since, was of the faculty of one or both, successively, of the colleges of Savannah; and since then of others too numerous to mention. He has always been an acceptable and eloquent teacher.

---

DIPLOMA TRANSLATION.—*The College Record* contains a translation of the Jefferson diploma. Why not print it in English at first, if it needs translation for the recipient? Or, why not append the translation on the same bit of parchment with the Latin? We once knew a preacher who, it is said, made a prayer in Latin and then translated it, lest the Lord might fail to appreciate his petition.

---

THE *British Medical Journal* reports an ear of corn discharged through the chest. The assailant must have used a cannon, or, at least, a pistol with an abnormally large meatus. But the "friend at our elbow" suggests that our British cousins (or grandparents) say *corn* when they intend *wheat*; and that the patient fired his own charge, after having thus loaded himself to the muzzle.

---

WE have received the first number of volume one of the *American Medical Digest*, edited by John C. Lester, A.M., M.D., and published monthly by H. Campbell & Co., New York.

It proposes to give a comprehensive digest of the most important articles that appear in American and English medical journals, and is designed especially for the "busy practitioner."

---

THE *Nashville Journal of Medicine and Surgery* presents a report of a case operated on at the clinic of Prof. W. T. Briggs, for large tumor of the neck, in which the "patients clothing, saturated with the ether, took fire from the spray producer, but the flames were quickly subdued and the operation proceeded with."

---

STATE REGULATED VICE.—It seems there is an "International Federation to Promote the Abolition of State-Regulated Vice" (or words to that effect). If the object is the abolition of the vice, we are ready to join. If it is merely for the abolition of the state-regulation, the question, we think, is still open for discussion.

---

THE Transactions of the Medical Association of Georgia,

1881, has been received from the Secretary, Dr. A. Sibley Campbell, of Augusta. It is a handsome volume; and will receive further notice in our next issue.

---

THE Early Struggles of Jefferson Medical College is an interesting paper presented in the November number of the *College and Clinical Record*, published in the interest of that school.

---

DR. J. MARION SIMS is in Europe, in the southern part of France, for the present. It will prove a universal source of regret that failing health induced the journey.

---

ST. BARTHOLOMEW'S HOSPITAL, of London, is said to possess an income of $4,950,000. Here in Atlanta we ask, can such things be? And answer, not in this city.

---

DR. ISAAC I. HAYES, the Arctic explorer and surgeon of Dr. Kane's expedition (second Grinnell), died in New York, from heart disease, Dec. 17, 1881.

---

TEXAS voted to locate the medical department of her State University at Galveston. Austin gets the literary part of the institution.

---

ST. LOUIS MEDICAL AND SURGICAL JOURNAL.—This publication completed its forty-first volume with the December number.

---

THE College of Physicians and Surgeons is the name of the new one with which Chicago was last attacked.

---

PROF. JNO. T. HODGEN, of St. Louis, is to publish his lectures on fractures and dislocations.

---

WELL GROWN.—The Medical Society of the County of New York has over eight hundred members.

